# Arthroscopic-assisted Arthrodesis of the Knee Joint With the Ilizarov Technique: A Case Report and Literature Review

**DOI:** 10.1097/MD.0000000000002540

**Published:** 2016-01-22

**Authors:** Michal Waszczykowski, Kryspin Niedzielski, Maciej Radek, Jaroslaw Fabis

**Affiliations:** From the Department of Arthroscopy, Minimally Invasive Surgery and Sports Traumatology, Medical University of Lodz, Poland (MW, JF); Department of Neurosurgery and Peripheral Nerve Surgery, Medical University of Lodz (MR); Department of Pediatric Orthopaedy and Traumatology, Polish Mother's Memorial Hospital Research Institute, Lodz, Poland (KN).

## Abstract

Arthrodesis of the knee joint is a mainly a salvage surgical procedure performed in cases of infected total knee arthroplasty, tumor, failed knee arthroplasty or posttraumatic complication.

The authors report the case of 18-year-old male with posttraumatic complication of left knee because of motorbike accident 1 year before. He was treated immediately after the injury in the local Department of Orthopaedics and Traumatology. The examination in the day of admission to our department revealed deformation of the left knee, massive scar tissue adhesions to the proximal tibial bone and multidirectional instability of the knee. The plain radiographs showed complete lack of lateral compartment of the knee joint and patella. The patient complained of severe instability and pain of the knee and a consecutive loss of supporting function of his left limb. The authors decided to perform an arthroscopic-assisted fusion of the knee with Ilizarov external fixator because of massive scar tissue in the knee region and the prior knee infection.

In the final follow-up after 54 months a complete bone fusion, good functional and clinical outcome were obtained.

This case provides a significant contribution to the development and application of low-invasive techniques in large and extensive surgical procedures in orthopedics and traumatology. Moreover, in this case fixation of knee joint was crucial for providing good conditions for the regeneration of damaged peroneal nerve.

## INTRODUCTION

Arthrodesis of the knee joint is an initial or salvage surgical procedure, which must be fully accepted by the patient. In cases of infected total knee arthroplasty, posttraumatic complication, tumor, failed knee arthroplasty or instability, it provides the opportunity to restore the patient's mobility and improve the quality of life. Today, many methods are used for arthrodesis of the knee, including intramedullary nailing, external fixator, or dual plating. The choice of the method depends on many factors: the cause of arthrodesis, the general condition of the patient and the condition of the body area to be operated, as well as past or ongoing infections and the age of the patient. In special cases, including knee infection or large skin defect, arthroscopic-assisted arthrodesis may be performed. The article presents a case of a young man who underwent an arthroscopic-assisted arthrodesis of the knee joint with the Ilizarov technique because of posttraumatic complications of the knee joint.

### Informed Consent

The patient was informed about all aspects of the chosen treatment, and his informed consent was given for the treatment. Written informed consent also was obtained from this patient for subsequent submission for publication and for all images. In this case a separate consent by local Ethics Committee was not required.

## CLINICAL REPORT

**FIGURE 1:**
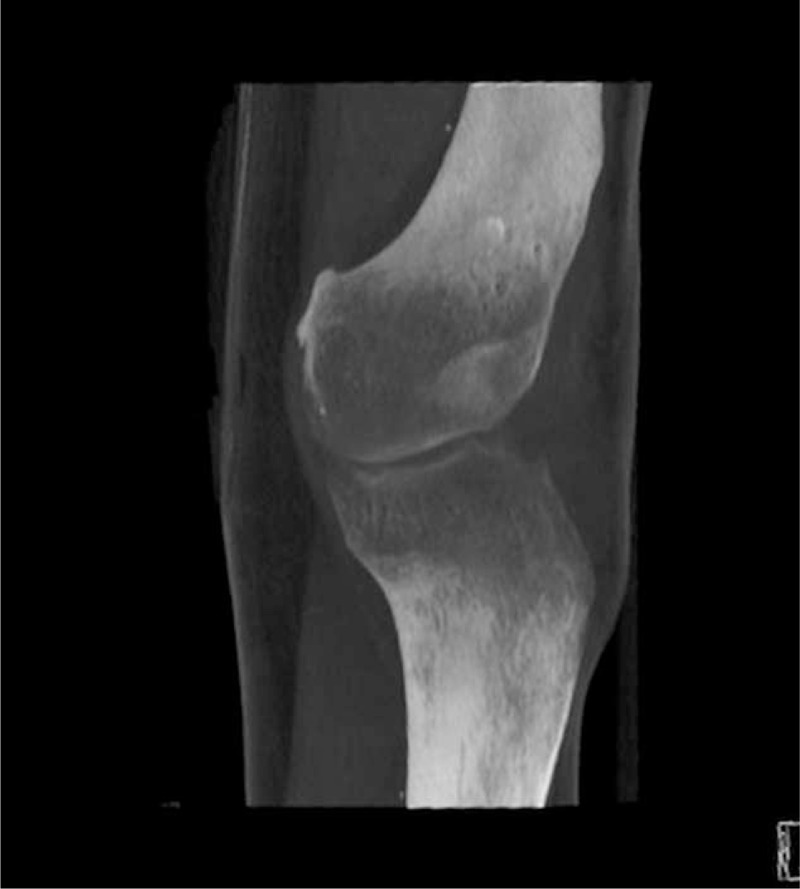
The computed tomography AP scan of the knee before arthrodesis.

**FIGURE 2:**
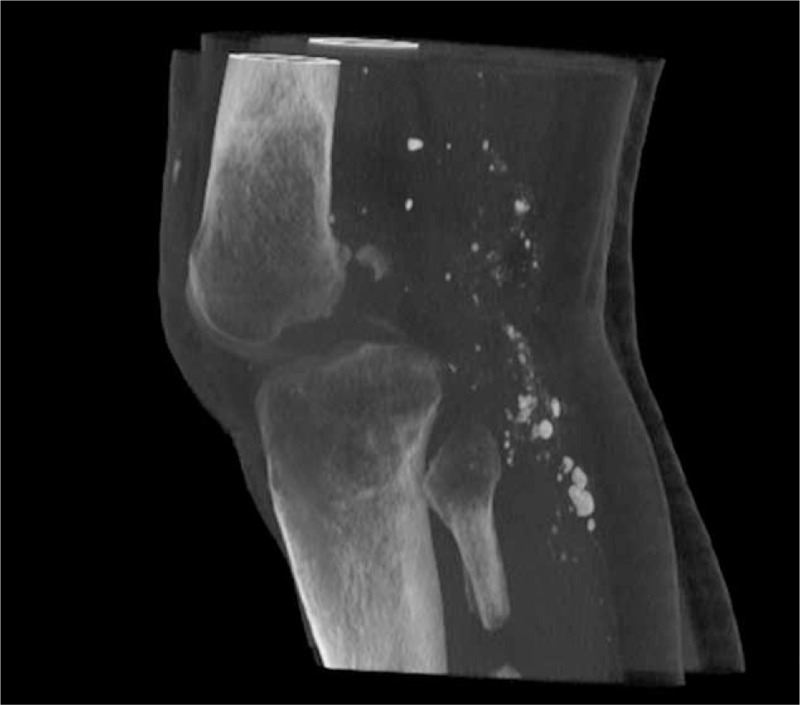
The computed tomography lateral scan of the knee before arthrodesis.

**FIGURE 3:**
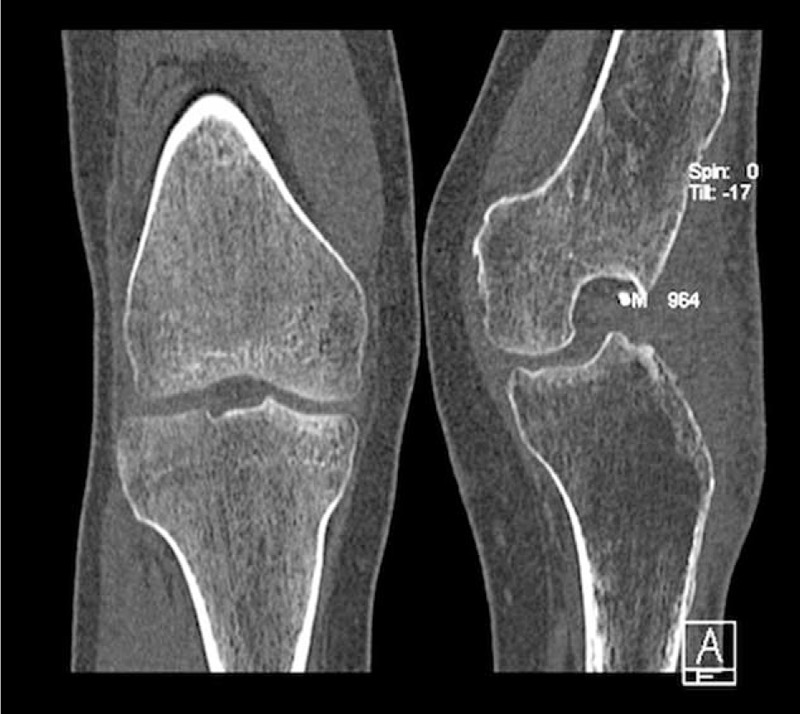
The computed tomography AP scan of both knees before arthrodesis.

A young patient aged 18 was admitted to the Department of Arthroscopy, Minimally-Invasive Surgery and Sports Traumatology 12 months after a multitissue injury of the left knee in a motorbike accident. Examination revealed an extensive lacerated wound in the knee area, a huge skin defect, type 33-B1/41-B3 fracture (according to Arbeitsgemeinschaft fur Osteosynthesfragen Foundation and American Orthopaedic Trauma Association Classification of Fractures and Dislocations—Arbeitsgemeinschaft fur Osteosynthesfragen Foundation/American Orthopaedic Trauma Association Classification of Fractures and Dislocations) of lateral femoral and tibial condyle with their amputation, comminuted fracture of the patella, an injury of the popliteal artery and veins, as well as palsy of the peroneal nerve (Figures 1 to 3). The patient was operated on in the local Department of Orthopaedics and Traumatology immediately after the accident. During the surgical procedure, the wound was thoroughly cleaned, necrotic tissues were removed, the damaged arterial and venous vessels of the limb were reconstructed and the peroneal nerve was decompressed. In the early perioperative period, an infection of the wound and skin necrosis was observed, which required further pharmacological and surgical treatment. In the early perioperative period, an infection of the wound, skin, and the other tissue necrosis was observed. The necrectomy of the affected tissue was necessary and resection of the patella was done. The multistage procedure of skin graft was needed to close and heal the wound. The multidrug antibiotic therapy was also performed.

On admission to our department, clinical examination confirmed extensive scar tissue lesions in the area of the proximal tibia and distal femur, as well as a deformation of the outlines of the knee caused by both scar tissue and amputation of lateral compartment of the joint. A physical examination confirmed considerable anterior and lateral instability, demonstrated by anterior drawer test (+++), positive Lachman test (+++), and positive pivot-shift test (+++). Limited flexion of the joint up to the maximum flexion of 90° with a full extension in the knee was also noted. No signs of a recent infection, inflammatory lesions or fistulas were observed on the skin surface in the knee region.

Physical examination of the ankle joint revealed signs of peroneal nerve paralysis with fixed 15° plantar flexion, and passive 25° dorsal flexion. The patient, however, did not demonstrate an active movement of the dorsal flexion in this joint. Physical examination of the hip joint in the left lower limb did not confirm any changes or limited mobility. Radiographic examination (x-ray) showed a complete lack of the lateral femoral and tibial condyle after total patellectomy.

Limb dysfunction, caused by instability and pain in the knee joint, led to the loss of its supporting function, which demanded the use of forearm crutches. Hence, surgery was planned to improve stability in the joint and restore the supportive function of the limb. Owing to the cause and degree of the instability, the decision to perform an arthrodesis of the knee was made. The presence of extensive skin lesions and scar tissue adhesion to the bone required an arthroscopic-assisted arthrodesis of the knee joint to be performed by the Ilizarov technique.

**FIGURE 4:**
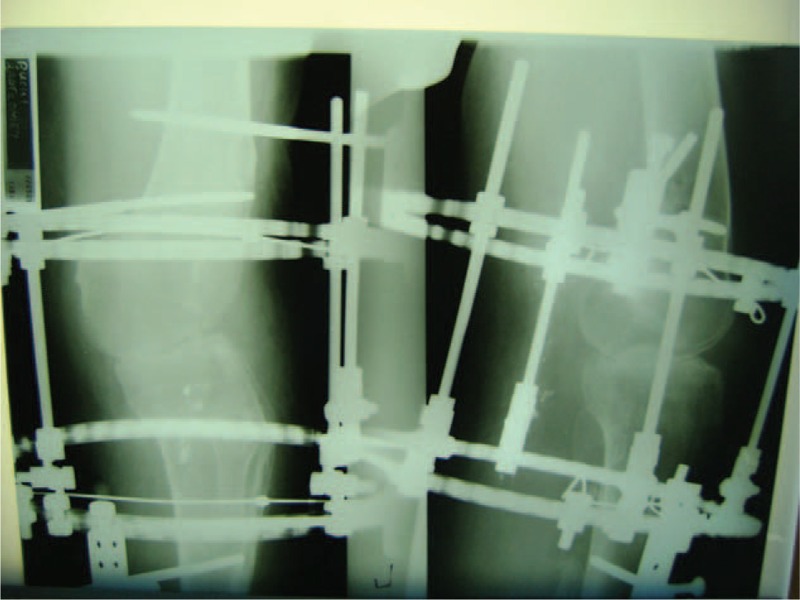
The x-ray of the left knee 2 days after the surgery.

**FIGURE 12:**
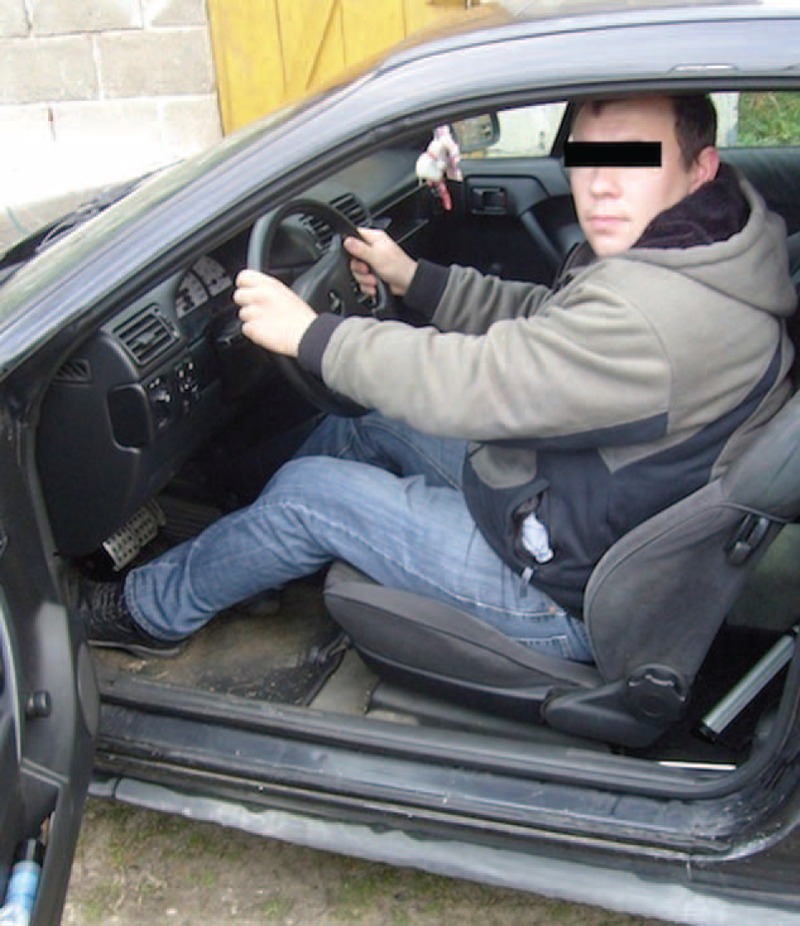
Two years after the surgery. The patient returned to work.

The surgery began with an arthroscopy. A complete lack of the lateral femoral and tibial condyle and lateral meniscus were observed. The anterior cruciate ligament was not found either. A proliferated inflamed synovium was noted in the joint. On the medial condyle of the femoral bone, signs of chondromalacia with the lesion of I°/II° in the weight-bearing region were observed. The inflamed synovium was then removed using a shaver and bipolar electrode. Next, with the use of a cochlea and the shaver, the articular cartilage was removed from the medial condyle of femoral bone, revealing the subchondral layer with signs of slight bleeding. A similar procedure was performed on the medial tibial plateau. Next, a total medial meniscectomy was performed. Fluoroscopy was used as guidance to fix the Ilizarov external fixator, with the knee flexed in 15° (Figure 4). At the end of the procedure, an autologous growth factor was administered to the knee (Gravitational Platelet Separation III System, Biomet Biologics, Inc. Warsaw, Indiana, USA). During the 2-week period after the operation, flexion of the knee was changed to 30° at the request of the patient; being a taxi driver, he needed to remain in sitting position during work (Figure 12).

**FIGURE 5:**
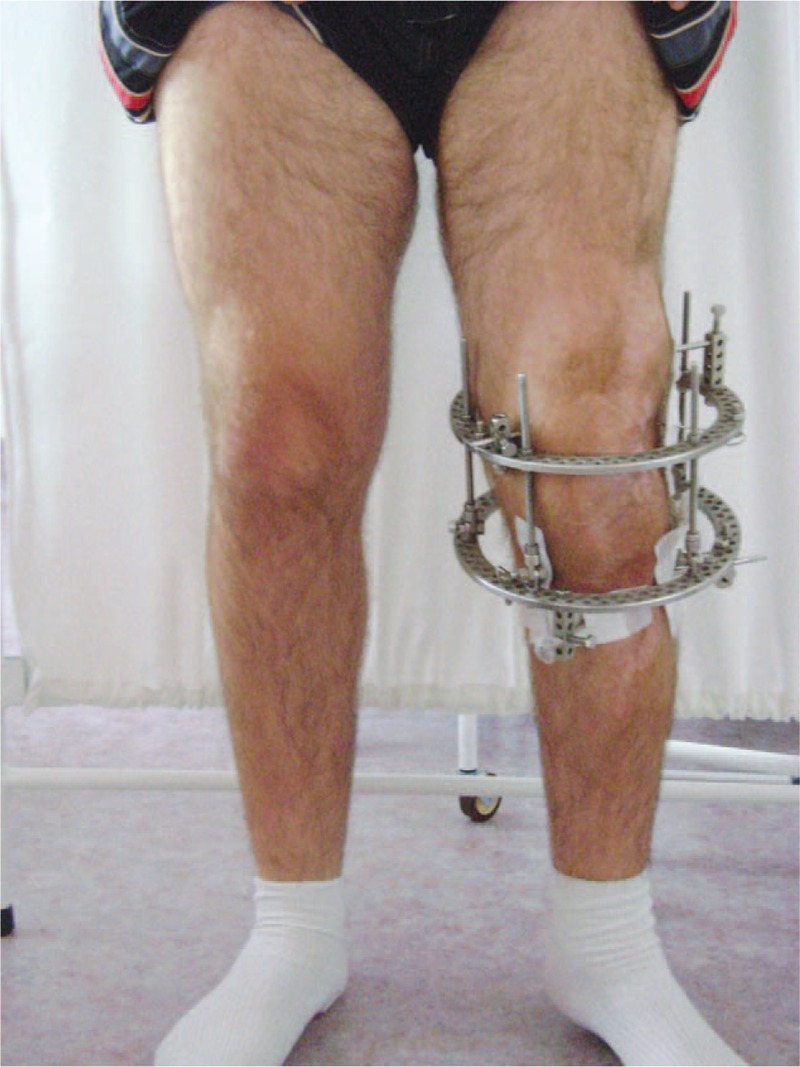
Ten days after arthrodesis.

**FIGURE 6:**
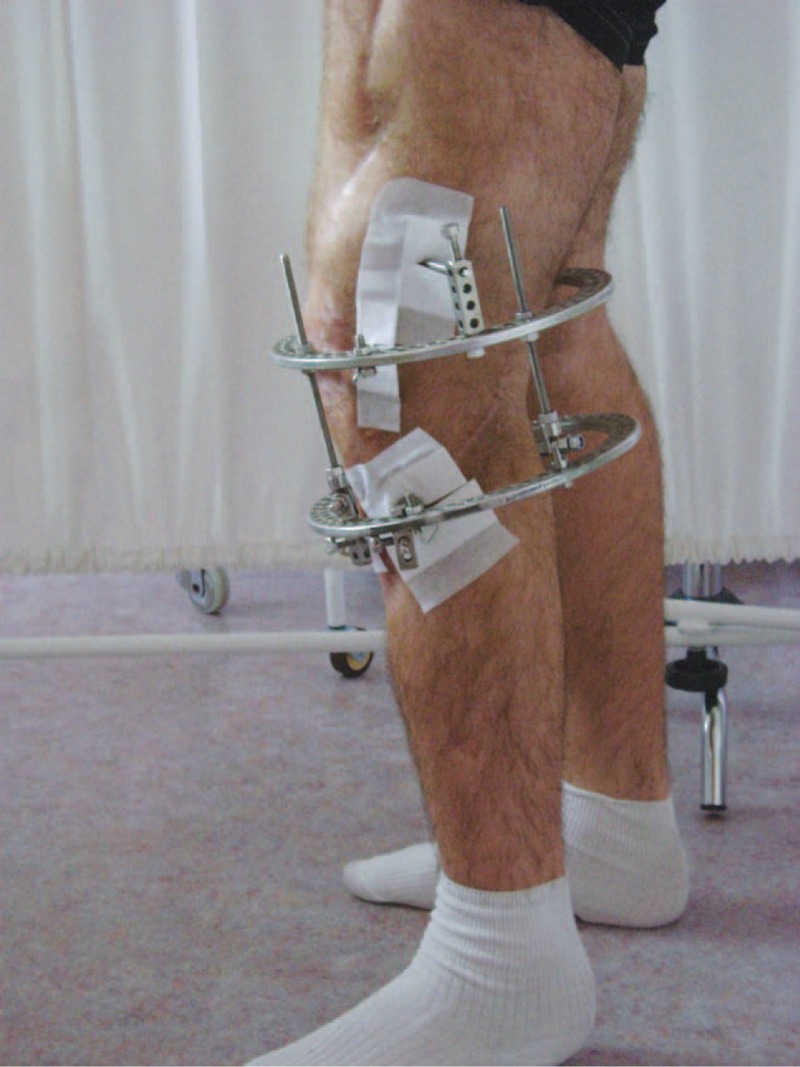
Ten days after arthrodesis.

**FIGURE 7:**
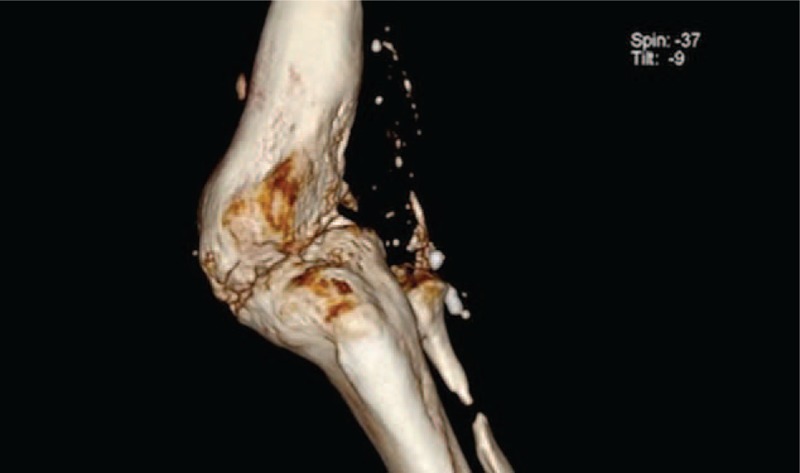
The computed tomography three-dimensional scan with signs of bone fusion.

**FIGURE 8:**
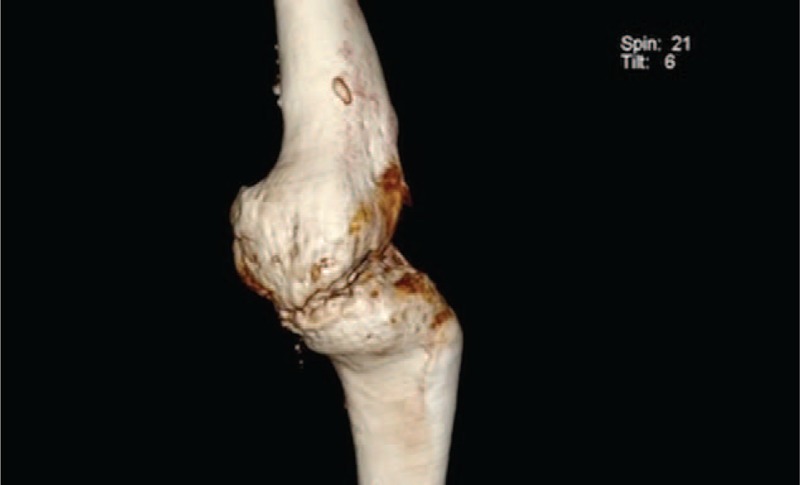
The computed tomography three-dimensional scan with signs of bone fusion.

**FIGURE 9:**
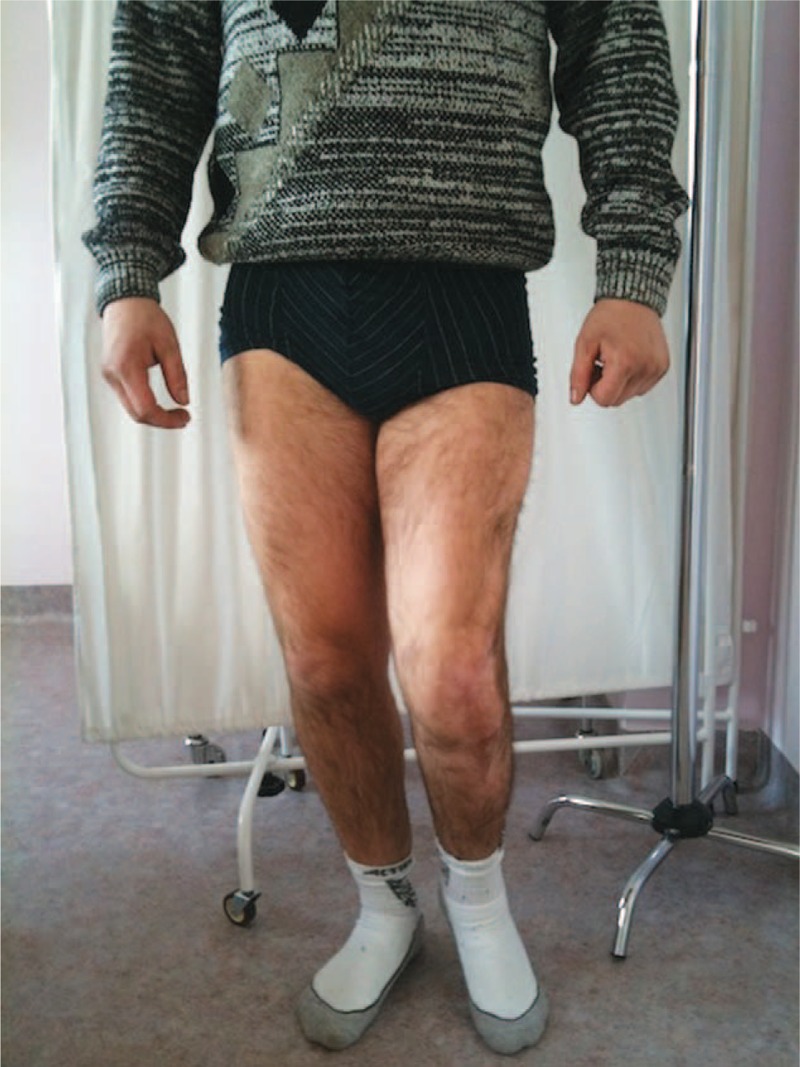
Fifty-four weeks after the surgery.

**FIGURE 10:**
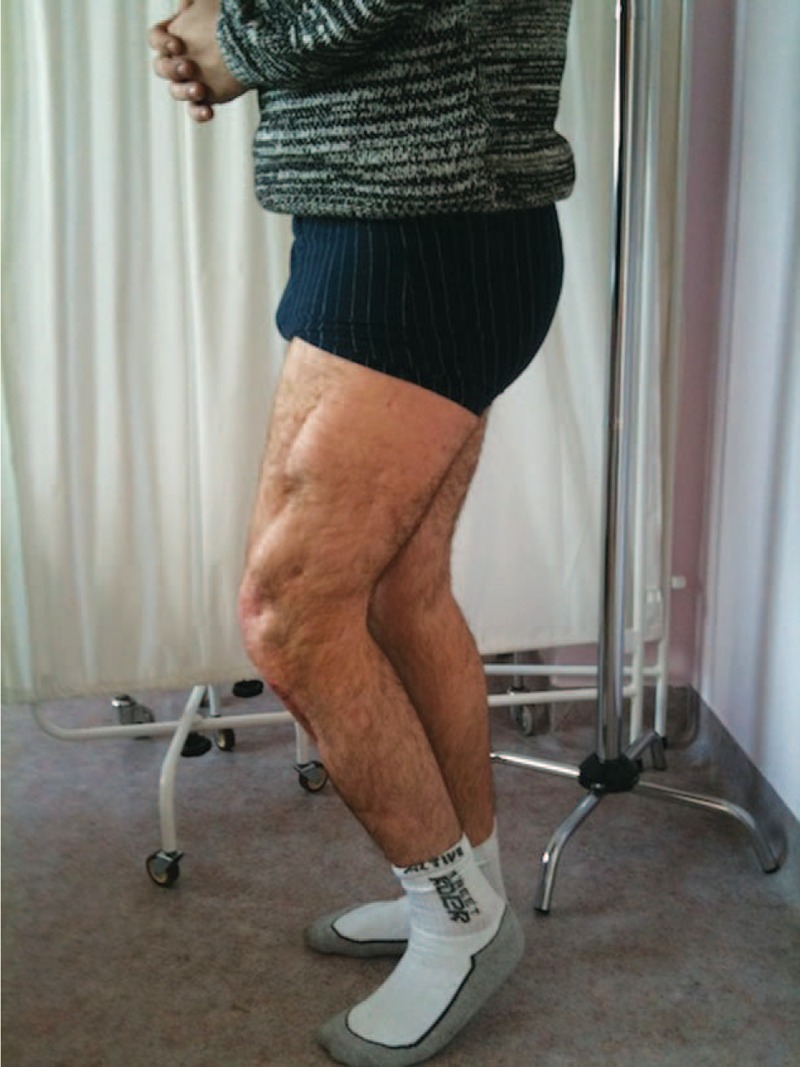
Fifty-four weeks after the surgery.

The limb remained immobilized in the Ilizarov apparatus for a total period of 42 weeks (Figures 5 and 6). Owing to persistent pain in the arthrodesis region, it was decided to immobilize the limb in a plaster cast for the next 6 weeks and then in a plaster splint for another 6 weeks. At the end of this period, check-up computed tomography was performed to confirm complete bone fusion (Figures 7 and 8). After the surgery, the patient walked with forearm crutches. He was advised to avoid putting his full weight on the limb for the first 2 weeks, although partial and gradual weight bearing was permitted after this period. Finally, after a period of 6 weeks, full weight bearing was recommended (Figures 9 and 10). Follow-up radiologic examinations were performed soon after the surgery, then after 3 weeks and every 6 weeks until the removal of the apparatus. Radiographic examinations were assessed by independent radiologists. The subsequent examinations confirmed gradual bone fusion. Over the whole immobilization period, no major infections were noted in the area treated with the Ilizarov technique or any signs of inflammatory processes in the joint or bones.

## DISCUSSION

The article presents a difficult case of young man with significant, posttraumatic dysfunction of his lower extremity, preventing him from normal and independent life. Decision to undertake the treatment and perform knee arthrodesis was difficult, but necessary. It was determined that the procedure provided the only chance for the patient to regain fitness and return to daily activity and work. Additional circumstances would complicate traditional technique of knee arthrodesis. Owing to local status of skin and soft tissues in the knee area, decision was made to perform the procedure by means of low-invasive arthroscopy and external fixation with Ilizarov apparatus. Although arthrodesis of the knee joint is a last-choice surgical procedure, this procedure is sometimes the only possibility to restore the supporting function of the limb and improve the patient's quality of life. Arthroplasty of the knee resulting in infection, septic loosening of the joint endoprosthesis, and postinfectious complications are indications for initiating an arthrodesis.^1–5^ Posttraumatic complications of the knee joint are infrequent indications for its primary immobilization.^6–7^ The justification in the case of the 19-year-old boy presented in the article was a combination of the multidirectional instability of the left knee joint, caused by amputation of the lateral compartment of the joint, and a multiligamentous injury. No information on the use of the knee joint arthrodesis in the treatment of such posttraumatic complications is given in the literature.

An important problem in the initiated therapy was a choice of immobilizing techniques in this patient. In most cases an arthrodesis of the knee joint involves fixation with the use of intramedullary nails, plates, screws and external fixators.^3,4,8,9^ The most effective procedure appears to be the one that uses intramedullary nails, with an estimated efficacy between 88% and 100%.^8–14^ There, however, are certain limitations of this method and it should not be applied in the case of recent infections and bone inflammation. The dual plating method is only slightly less effective but involves more extensive body area, which might contribute to traumatization of tissues and increase the risk of infections.^13,15,16^ External fixators appear to be least effective.^17^

**FIGURE 11:**
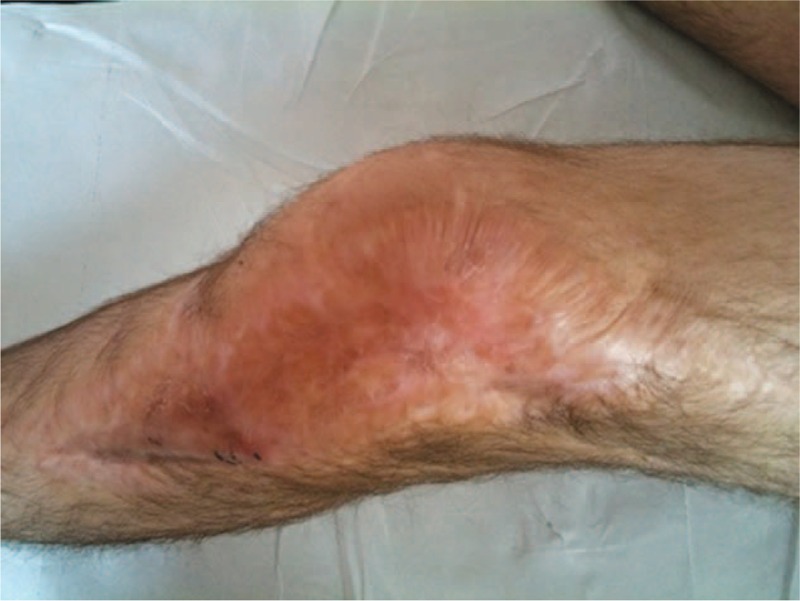
Large skin defect of the knee region.

Between 64% and 100% of the patients treated with the Ilizarov technique demonstrated a successful arthrodesis.^18–19^ This kind of fixation is almost a noninvasive method of immobilizing of a joint. It is important if the patient has previously suffered from *Stapylococcus aureus* infection.^12,20,21^ The Ilizarov apparatus allows the compression of the fixed elements to be maintained and controlled, provides stabilization, allows early weight bearing and it can be applied even during an ongoing infection.^21,22^ Extensive cicatricial skin lesions observed in the knee area were an indication for the selection of this particular method in the current case (Figure 11). Numerous surgical scars with signs of keloids, combined with the poor condition of the surrounding skin seen in the patient, might have caused complications in the healing process, and these factors provided indications for arthroscopic-assisted arthrodesis. The removal of the articular cartilage and preparation of the articular surface for fusion with the use of an arthroscope prevented the poor skin from being surgically invaded, which reduced the traumatization of soft tissues.

Little data exists concerning this method of knee arthrodesis.^6,7^ Papillon et al report the effectiveness of the arthroscopic technique in performing an arthrodesis of the knee joint in a young female patient with posttraumatic gonarthrosis, which resulted in infectious complications.^6^ The application of this noninvasive technique was associated with reduced risk of infections and traumatization of tissues. Also Acquiter et al confirmed the effectiveness of arthroscopic-assisted arthrodesis of the knee joint with a single-axis external stabilizer; complete bone healing was observed 5 months after immobilization in all the patients.^7^

In the case described above, the flexion of the knee was changed to the 30° at the patient's request for 2 weeks after the operation. The patient was a taxi driver and he needed to stay and work in sitting position (Figure 12).

In the presented case study, a gradual restoration of the function of the peroneal nerve, also seen in electromyographic evaluation, was a crucial process. The restoration of the joint stability was highly important as it contributed to the process of healing and regeneration of the nerve. A gradual improvement was observed of the function of the muscles located in the area innervated by peroneal nerve, as well as a restoration of the superficial sensibility of the skin on the dorsal side of the foot and tibia.

This article has its limitations. Only 1 case with good clinical and radiologic outcome was presented. Such significant posttraumatic dysfunctions of knee joint are not common and it is very hard to collect larger patient group. Thus, repeatability and efficacy of this surgical technique cannot be proved. This case, however, provides a significant contribution to the development and application of low-invasive techniques in large and extensive surgical procedures in orthopedics and traumatology. Interdisciplinary approach is a clear advantage of this study.

Literature review performed involved publications raising the issues of significant, posttraumatic dysfunctions of knee joint, septic complications of knee joint area following previous surgical procedures and application of external fixation in knee joint fusion procedures. Low-invasive nature of knee arthrodesis provided an additional criterion for the selection of literature for the review. Review involved source materials from PubMed, ProQuest, EBSCO, Embase (Wolters Kluver/OVID), ScienceDirect, etc. databases.

## CONCLUSIONS

The presented case of an arthroscopic-assisted arthrodesis of the knee joint confirms that this technique might be a safe and effective method for achieving knee joint fusion in patients with contraindications for open procedure is not possible. This method can be considered in patients with past infections of bones and the knee joint because it is almost minimally invasive and its application hardly ever results in perioperative infections. It also demonstrates the important role played by arthrodesis of the knee for restoring the stability of the knee and improving the function of the nerve in cases with concomitant nerve palsy.
